# Holographic Lenses in an Environment-Friendly Photopolymer

**DOI:** 10.3390/polym10030302

**Published:** 2018-03-11

**Authors:** Tomás Lloret, Víctor Navarro-Fuster, Manuel G. Ramírez, Manuel Ortuño, Cristian Neipp, Augusto Beléndez, Inmaculada Pascual

**Affiliations:** 1 Departamento de Óptica, Farmacología y Anatomía, Universidad de Alicante, Apartado de correos 99, Alicante E-03080, Spain; tomasll10@hotmail.com (T.L.); ramirez@ua.es (M.G.R.); 2 Departamento de Física, Ingeniería de Sistemas y Teoría de la Señal, Universidad de Alicante, Apartado de correos 99, Alicante E-03080, Spain; victor.navarro@ua.es (V.N.-F.); mos@ua.es (M.O.); cristian@ua.es (C.N.); a.belendez@ua.es (A.B.)

**Keywords:** holographic lenses, environment-friendly photopolymer, volume holography

## Abstract

In this paper, we theoretically and experimentally evaluated the quality of volume phase transmission lenses stored in an environmentally friendly photopolymer. Holographic lenses (HLs) were obtained using symmetrical and asymmetrical experimental setups with the same positive and negative focal length and pupil diameter. The image quality was evaluated from the calculation of the modulation transfer function (MTF) by capturing the point spread function (PSF) with a charge-coupled device (CCD). A maximum frequency of 14 L/mm, reaching an MTF value of 0.1, was obtained for a negative asymmetrically recorded HL, evaluated at 473 nm wavelength. A theoretical study of aberrations was carried out to qualitatively evaluate the experimental results obtained.

## 1. Introduction

Holography is based on Gabor’s principle of wavefront reconstruction [1]. It is a technique to register and reconstruct three-dimensional objects. Holographic optical elements (HOEs) are among the most interesting applications of holography because they replace curved and heavy refractive optical elements with a simple, flat, and lightweight element.

HOEs store the interference pattern produced by two spatially overlapping coherent beams. This pattern creates a photonic structure capable of diffracting light in a desired way.

The first HOE concept of holographic application, a holographic mirror, was described by Denisyuk in 1962 [2]. A point-source hologram which acts as a lens was demonstrated by Schwar et al. in 1967 [3]. It is possible to gather various functions in a single substrate by multiplexing two or more HOEs [4,5] according to its high diffraction efficiency and narrow-band frequency characteristics. 

HOEs are widely used in many fields, such as the relief thin holograms used in credit cards or volume holographic optical elements that function as phase index modulation holograms seen in holographic projections, couplers, storage, filters, and displays [6,7,8,9,10]. 

HOEs have evolved in parallel to the development of photonics, communications, and optical information processing. Innovative technologies demand versatile and environmentally compatible optical elements. Holographic lenses in particular show enormous potential in highly relevant applications, such as in virtual and augmented reality head-mounted displays as image systems [11,12,13] or in light deflectors and concentrators as non-image systems [14,15,16].

The key points of this applicability are the new recording materials, able to perform under the most specific situations and applications. 

Typically, different materials, such as silver halide emulsion [17,18], dichromated gelatin [19,20], photoresist [21], photorefractive [9], or photopolymer [22] are used in the manufacturing of HOEs. Photopolymers were first used as holographic optical elements by Close et al. in 1969 [23]. Since then, the range of photopolymer materials in optical applications has grown enormously [24]. This is mainly due to their versatility both in composition and in design as well as to other interesting properties, such as self-processing capabilities [25], low cost, good dimensional stability, variable thickness, high energetic sensitivity, large dynamic range, sharp angular selectivity, and flexibility. In this sense, the importance of photopolymers is growing spectacularly [26].

However, commonly used hydrophilic photopolymers contain poly(vinyl alcohol) (PVA), gelatin binders, or monomers related to acrylamide [27,28,29,30]. This type of photopolymers has certain undesirable features, such as the toxicity of some of its components; for instance, acrylamide has a high potential to cause cancer. In order to avoid this risk, the latest trends in photopolymers include materials with low toxicity [31,32,33] and exclude traditional solvents for better environmental compatibility [34,35]. Additionally, these types of materials have good recycling properties. In this sense, we developed “Biophotopol”, an environmentally friendly photopolymer for use as a recording holographic material in optical applications [4,36,37,38].

In this work, we evaluated holographic lenses (HLs) fabricated in Biophotopol as an image system. In this case, two types of HLs were fabricated, symmetrical and asymmetrical, each with the same distance focal length. For each type, we registered both positive and negative distance focal lengths to evaluate the image quality of the lenses with the environmentally friendly Biophotopol material.

## 2. Materials and Methods

### 2.1. Preparation of the Material

**Figure 1:**
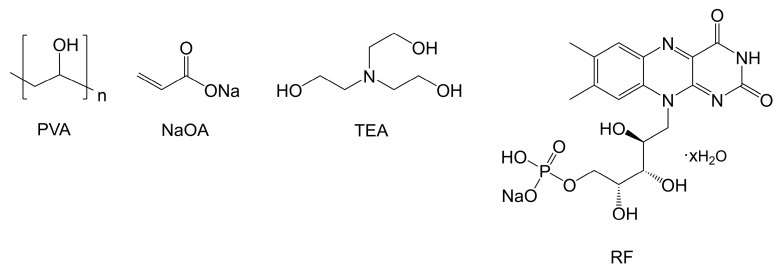
Chemical structures of the prepolymer components.

Volume phase transmission HLs were recorded in the environmentally friendly photopolymer Biophotopol. Different compositions of this photopolymer were studied in our previous papers [36,37,38]. In the present work, the prepolymer solution was composed of poly(vinyl alcohol) (PVA) as inert binder polymer (*M*_w_ = 130,000 g/mol, hydrolysis grade = 87.7%), sodium acrylate (NaAO) as polymerizable monomer, triethanolamine (TEA) as coinitiator and plasticizer, and sodium salt 5’-riboflavin monophosphate (RF) as sensitizer dye. The NaAO was generated in situ through a reaction of acrylic acid (HAO) with sodium hydroxide (NaOH) in a 1:1 proportion. All compounds were purchased from Sigma-Aldrich Quimica SL (Madrid, Spain). The solvent was water, in which all components were soluble. The chemical structures are shown in Figure 1. The optimized concentrations in the prepolymer solution were 13.5 *w/v* %, 0.39 M, 9.0 × 10^−3^ M, and 1.0 × 10^−3^ M for PVA, NaAO, TEA, and RF, respectively.

The prepolymer solution was deposited through force of gravity on a leveled glass plate (6.5 × 6.5 cm^2^), which had been previously washed and dried and left inside an incubator (Climacell 111, MMM Medcenter Einrichtungen GmbH, Munich, Germany) with controlled conditions (relative humidity = 60% ± 5% and temperature = 20 ± 1 °C) for 24 h. The process was carried out under controlled light conditions to which the material was not sensitive. A part of the water evaporated until reaching equilibrium with the environmental conditions inside the incubator during the drying time. At that time, the layers were then ready for recording, which took place immediately. After the exposure and reconstruction, the thicknesses of the dried layers were measured with an ultrasonic pulse-echo gauge (PosiTector 200, DeFelsko, Ogdensburg, NY, USA). The physical thickness of the photopolymer layer was around 200 µm. To avoid damaging the lenses, the thickness was measured over unexposed areas. In order to increase the stability, the holographic lenses were cured with a LED lamp (13.5 W, 875 lumens at 6500 K, Lexman, Alicante, Spain) for 20 min. The residual dye was eliminated during this process. 

It should be noted that the TEA concentration in this work varied with respect to the previous Biophotopol composition. The optimization of TEA concentration in the prepolymer solution was very important because of its function as both a coinitiator and a plasticizer. TEA lowered the glass transition temperature of the photopolymer, maintaining the dimensional stability of the dry layer under standard environmental conditions. Another important function of TEA was its ability to increase the thickness of the photopolymer layer because of its high boiling point, which was accompanied by a decrease in the concentration of the photopolymer components. This factor had to be taken into account when the concentrations of the components present in the photopolymer layer were optimized. Furthermore, TEA affected the viscosity of the medium, increasing the diffusion coefficients of the components as the concentration of TEA became greater. However, taking into account the capacity of TEA to form a H−bond with water, a low content of TEA made it more difficult for the NaAO to dissolve, leading to crystallization during the drying process. The environmental conditions during the recording stage had to be controlled to avoid precipitation of NaAO on the surface of the photopolymer layer. Also, the permeability of PVA played an important role in the drying and recording stages. Therefore, the control of humidity, temperature, and TEA/water ratio in the dry layer with respect to the thickness and final composition of the photopolymer layer was very important to obtain high diffraction efficiency.

### 2.2. Holographic Setup

**Figure 2:**
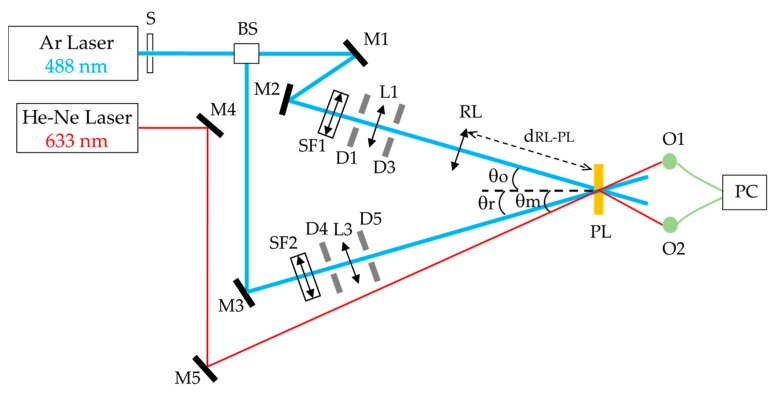
Experimental setup for recording of holographic lenses. S: electronic shutter, BS: beam-splitter, Mi: mirrors, Li: lenses, Di: diaphragms, SFi: spatial filters, RL: refractive lens, PL: photopolymer layer, Oi: optical power meters, PC: data recorder.

The experimental holographic setup used to record HLs is shown in Figure 2a. An Argon laser beam tuned at 488 nm wavelength, at which the material is sensitive, was split into two secondary beams, the reference and object beams, using a beam-splitter (Newport, Irvine, CA, USA). Both beams were spatially filtered and collimated. Subsequently, the object beam passed through a refractive lens (LR), emerging as a convergent beam. The two beams were recombined at the photopolymer layer with different incident angles (θ_o_ and θ_r_) to the normal of the photopolymer layer. The ratio of intensities between both beams was 1:1, and the total recording intensity was 3 mW/cm^2^. This value is the sum of both intensity beam measures in the hologram plane, adjusting the intensities in each case. The exposure time was 20 s. Because of the interference from both beams, bright (constructive interference) and dark (destructive interference) zones were produced in the photopolymer layer. In the bright zones, a radical polymerization reaction took place, and a modulation of the refractive index was generated. H. Peng et al. provided the mechanism for this reaction. When the photopolymer layer was illuminated, the RF absorbed photons, leading to the singlet excited state (^1^RF*). By means of intersystem crossing, the triplet excited state (^3^RF*) was generated. Subsequently, the ^3^RF^*^ was quenched by TEA through an electron transfer reaction, and TEA radicals were generated which were combined with acrylate monomers to generate chain initiators [39]. 

The focal length of the HL was obtained as:

*f’*_HL_ = *f’*_RL_ − *d*_RL-PL_, (1)

where *f’*_HL_ and *f’*_RL_ are the focal lengths of the HL and RL, respectively, and *d*_RL-PL_ is the distance between the RL and the photopolymer layer. In this work, positive (+) and negative (−) HLs were stored symmetrically and asymmetrically, with focal lengths of +90 and −90 mm at a wavelength of 488 nm. Since the photopolymer did not present any absorption, a He–Ne laser at 633 nm was used to visualize in real time the interference pattern registered in the HL at an angle θ_m_, indicated in Table 1. A summary of the incidence angles, focal lengths, and *d*_RL-PL_ used is presented in Table 1. It must be noted that the experimental θ_m_ (45°) for asymmetrical recording geometry differs from the theoretical value (46.8°). When the record of the HL was carried out in this geometry, changes in the orientation and spacing of the fringes were produced because of the shrinkage of the photopolymer layer [40,41,42] giving place to that difference. The shrinkage value was about 2.9%, and the index modulations was 0.001 [4].

### 2.3. Evaluation of Holographic Lenses

**Figure 3:**
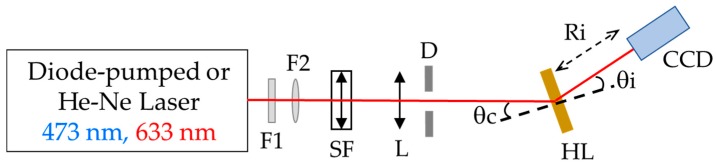
Experimental setup for the evaluation of holographic lenses. Fi: filters, SF: spatial filter, L: lens, D: diaphragm, HL: holographic lens, CCD: charge-coupled device.

The HLs were evaluated according to the quality of the images produced by them using the experimental setup shown in Figure 3. A diode-pumped laser emitting at 473 nm (near to recording wavelength) and a He–Ne laser at 633 nm were used to illuminate the HLs. The beam was spatially filtered and positioned at the reconstruction angle (θ_c_) which was equal to the recording angle. The conjugated beam was used when the evaluated lenses were negative. Images of the focal point image, located at a distance *R*_i_ and angle θ_i_ from the HL, were captured with a CCD camera (Thorlabs GmbH, Munich, Germany). Table 2 shows the reconstruction distance (*R*_i_) from the sample and the reconstruction (θ_c_) and image (θ_i_) angles. The experimental and theoretical values of the modulation transfer function (MTF) and the aberrations were obtained to evaluate the image quality of the HLs.

#### 2.3.1. MTF Calculation

The MTF measures the resolution of the image contrast compared with that of the object. For a perfect optical system without aberrations, in which only the limit imposed by the diffraction is taken into account, and considering a circular aperture, the theoretical MTF is given by W. Smith and C. García et al. [43,44]:

(2) $$MTF=\frac{2}{\pi }\left(\phi -cos\phi \cdot sin\phi \right)$$

where *ϕ* is obtained as:

(3) $$\phi =cos^{-1}\left(\frac{\lambda \upsilon }{2NA\prime }\right)$$

where *λ* is the wavelength measured in millimetres, *υ* is the frequency in lines·mm^−1^, and NA’ is the numerical aperture. The frequency at which the MTF becomes zero, the denominated cut-off frequency (*υ*_0_), is calculated as:

(4) $$\upsilon_{0}=\;\frac{2NA\prime }{\lambda }=\;\frac{1}{\lambda \left(f/\#\right)}$$

where *f/#* is the diaphragm number defined as:

(5) $$f/\#=\;\frac{f\prime }{\Phi_{\mathrm{PE}}}$$

where *ϕ*_PE_ is the entrance pupil of the lens that corresponds to the diameter of the lens.

The experimental MTF was obtained from the point spread function (PSF), which describes the response of a system to a point of light. The PSF was acquired through the images captured with a CCD Thorlabs DCU224 camera, which comprised a matrix of 1360 × 1420 pixel (horizontal × vertical) and a spacing between pixels (∆*x*) of 4.65 µm. For this value of ∆*x*, the Nyquist spatial frequency was 107.5 lines·mm^−1^. The image of a point is given by the diffraction spot for a perfect optic system. However, when the system contains aberrations, the PSF will depend on them. The PSF is the intensity distribution function in the image plane. The module of the optical transfer function (OTF), defined as the Fourier transform of the PSF, is the MTF. Through the representation of the theoretical and experimental MTFs, the cut-off frequencies can be determined for the HLs, considering them free of aberrations.

#### 2.3.2. Determination of the Aberrations

The aberrations of the HLs recorded were obtained theoretically in the exit pupil plane (PS) and in the image focal plane. According to J. Latta [45], the theoretical total aberration in the PS is calculated as:

(6) $$\Delta_{\mathrm{Total}}\;=\;\Delta_{S}+\;\Delta_{C}+\;\Delta_{A}$$

where *∆*_Total_ is the wave front deviation from the Gaussian sphere, and S, C, and A subscripts indicate the spherical, chromatic, and astigmatic aberrations, respectively. When the beams used in the recorded process of the HLs are in the same plane normal to the hologram, a simplification in the equations is possible. Thus, $\Delta_{S}$, $\Delta_{C},$ and $\Delta_{A}$ can be obtained as:

(7) $$\Delta_{S}\;=\;-\left(\frac{1}{8\lambda_{c}}\right){\left(x^{2}+\;y^{2}\right)}^{2}S,$$

(8) $$\Delta_{C}\;=\;\left(\frac{1}{2\lambda_{c}}\right){\left(x^{2}+\;y^{2}\right)}^{2}xC_{x},$$

(9) $$\Delta_{A}\;=\;-\left(\frac{1}{2\lambda_{c}}\right)x^{2}A_{x},$$

where *λ_C_* is the reconstruction wavelength. The *S*, *C*_x_, and *A_x_* are the aberration coefficients calculated as:

(10) $$S=\;\frac{1}{R_{C^{3}}}-\;\frac{1}{R_{i^{3}}}+\frac{\mu }{m^{4}}\left(\frac{1}{R_{O^{3}}}-\;\frac{1}{R_{R^{3}}}\right),$$

(11) $$C_{x}=\;\frac{sin\theta_{C}}{R_{C^{2}}}-\;\frac{sin\theta_{i}}{R_{i^{2}}}+\frac{\mu }{m^{3}}\left(\frac{sin\theta_{O}}{R_{O^{2}}}-\;\frac{sin\theta_{R}}{R_{R^{2}}}\right),$$

(12) $$A_{x}=\;\frac{sin^{2}\theta_{C}}{R_{C}}-\;\frac{sin^{2}\theta_{i}}{R_{i}}+\frac{\mu }{m^{2}}\left(\frac{sin^{2}\theta_{O}}{R_{O}}-\;\frac{sin^{2}\theta_{R}}{R_{R}}\right),$$

where *R*_i_ is the distance from HL to the Gaussian image, and θ_i_ is the image angle, i.e., the angle between the Gaussian image point and the plane containing the HL. *R*_R_ and *R*_C_ are the distances of the reference and reconstruction point sources, respectively, to the hologram plane. *R*_O_ is the distance between the object point and the HL, taking positive and negative values for HL+ and HL−, respectively. The µ factor denotes the wavelength’s shift *λ*_C_/*λ*_R_, and *m* is the factor by which the fringe spacing is increased or decreased (in this work we considered *m* = 1). Through Equations (10)–(12), the aberrations in the exit pupil plane can be obtained.

Additionally, according to C. García, et al. and J. Latta [44,45], the aberrations in the image plane can be calculated as:

(13) $$I\left(x^{\prime },y^{\prime };z\prime \right)=\frac{1}{B^{2}}{\left|\iint_{S}A\left(x,y\right)exp\left[i\Delta \left(x,y;x^{\prime },y^{\prime };z\prime \right)\right]dxdy\right|}^{2},$$

where *I(x’,y’;z’)* is the intensity in the image plane, *A(x,y)* = 1 for an amplitude uniform, *S* is the area of the exit pupil plane, and B is the amplitude at the Gaussian image point (*x*’ = *y*’ = 0) in the absence of aberration.

The experimental aberrations were obtained from the PSF captured with the CCD camera through the representation of the intensity profile. These results could be compared with those calculated theoretically by means of Equation (13).

## 3. Results and Discussion

The images’ quality of the holographic lenses was assessed both by experimentally obtaining the MTFs from the PSF images and by theoretically obtaining the intensity distribution in the image plane and the PS, as explained in Section 2.3.

### 3.1. MTF

The theoretical values for an MTF of 0.1 at 473 and 633 nm are 227 and 170 L/mm, respectively, from Equation (2).

**Figure 4:**
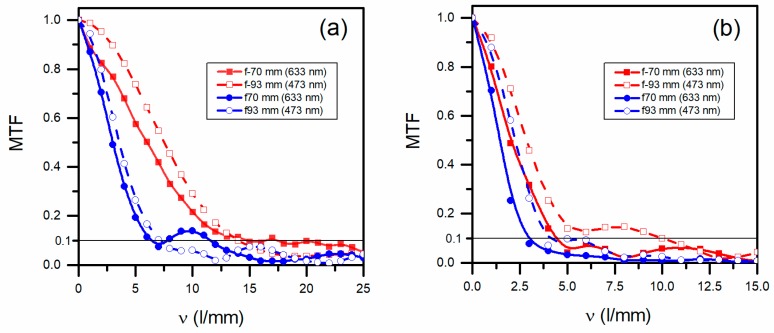
Experimental modulation transfer function (MTFs) for positive and negative HLs recorded (**a**) asymmetrically and (**b**) symmetrically. The frequencies of the HLs were determined for an MTF value of 0.1 (black, horizontal line).

The experimental MTFs of the HLs obtained for 473 and 633 nm wavelengths are shown in Figure 4. To better distinguish the MTF behaviour of the HL, we used the limiting resolution criteria of 0.1 [46]. At this limiting resolution, the maximum reached frequency of 14 L/mm was obtained for negative asymmetrically recorded HL, reconstructed at 473 nm wavelength (Figure 4a; red, dashed curve with empty squares). A similar positive asymmetric HL showed a frequency of 7 L/mm (Figure 4a; the blue, dashed curve with empty circles). Evaluating these asymmetrical HLs with a 633 nm wavelength showed a similar behaviour to that of their respective negative HLs at 473 nm wavelength. In Figure 4b, the evaluation of symmetric HLs showed a decrease in frequency at 0.1 MTF with respect to the asymmetric ones. The frequency difference between positive and negative symmetric HLs recorded was greater at different wavelengths. The frequencies evaluated at 473 nm were higher than at 633 nm. At the same time, the frequencies of the negative HLs were higher than their respective positive HLs.

In conclusion, the frequency at 0.1 MTF for asymmetrical HLs was similar for both wavelengths. For symmetrical HLs, it was higher for the wavelength of 473 nm. In addition, the HLs recorded asymmetrically had a higher spatial frequency at 0.1 MTF than the corresponding HLs recorded symmetrically.

### 3.2. Aberrations

A qualitative study of the aberrations was made, theoretically studying the aberrations produced by the HLs in the exit PS and in the focal image plane.

#### 3.2.1. PS Aberrations

**Figure 5:**
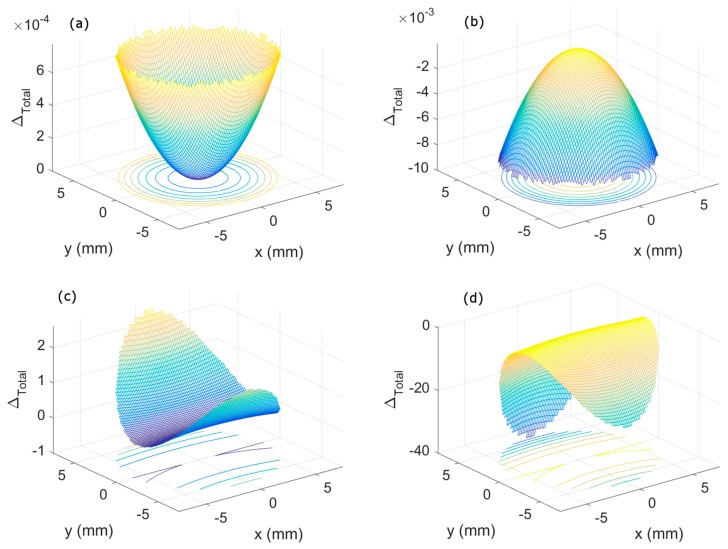
Total aberration (*Δ*_Total_) at the exit pupil plane (PS) of asymmetrical (**a**,**b**), and symmetrical (**c**,**d**), HLs recorded (**a**,**c**) at 473 nm and (**b**,**d**) at 633 nm wavelength.

For the study of theoretical aberrations of asymmetrically and symmetrically recorded HLs in the exit pupil plane, Equations (6)–(9) were used to obtain the intensity distribution at two wavelengths (Figure 5).

In the case of asymmetrical lenses, the total aberration was given by the spherical aberration (Figure 5a,b). When the wavelength used was 473 nm, the spherical aberration was positive (Figure 5a). When the wavelength used was 633 nm, the spherical aberration was negative. By using the 473 nm wavelength, the spherical aberration was one order of magnitude lower than at 633 nm.

In the case of symmetrical lenses, the total aberration was mainly given by astigmatism for both wavelengths. Comparatively, astigmatism at 473 nm was positive, whereas the astigmatism was negative at less than 633 nm.

#### 3.2.2. Image Plane Aberrations

**Figure 6:**
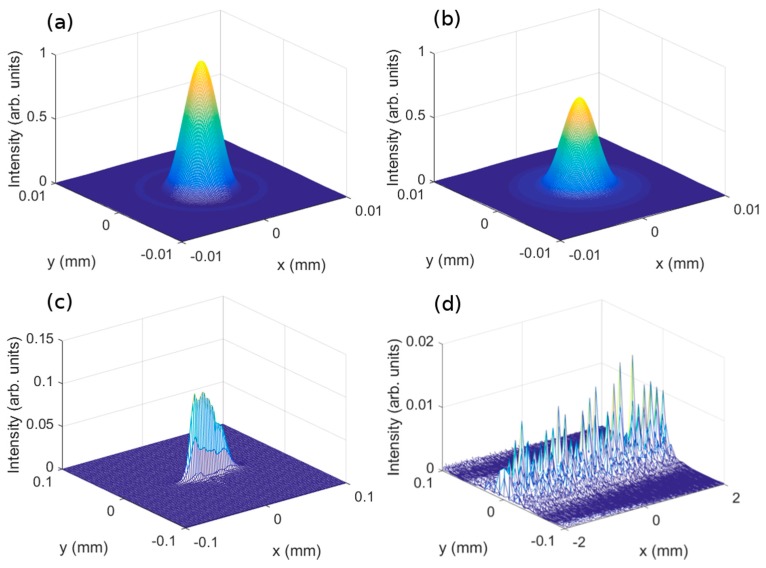
Intensity distribution at the image plane of asymmetrical (**a**,**b**), and symmetrical (**c**,**d**), HLs recorded (**a**,**c**) at 473 nm and (**b**,**d**) at 633 nm wavelength.

For the study of theoretical aberration in the image plane, Equation (13) was used to obtain the intensity distribution at two wavelengths (Figure 6).

For asymmetrical lenses, the intensity distribution corresponding to the optical system with a circular exit pupil and aberration-free intensity distribution was the airy pattern, centred at the Gaussian image point.

In the case of asymmetrical lenses, the image quality was limited only by the diffraction of the object radiation at the system’s exit pupil. At 473 nm wavelength (Figure 6a), the theoretical intensity of the image was maximum. However, at 633 nm, the theoretical intensity distribution in the image was slightly lower. For symmetrical lenses, the intensity distribution at the image point showed the astigmatism of the HL. At 473 nm wavelength reconstruction (Figure 6c), it was possible to see one of the Sturm focal lengths. In Figure 6d, the same HL reconstructed at 633 nm had the worst image quality. In all cases, it was possible to improve the image quality by decreasing the pupil diameter.

**Figure 7:**
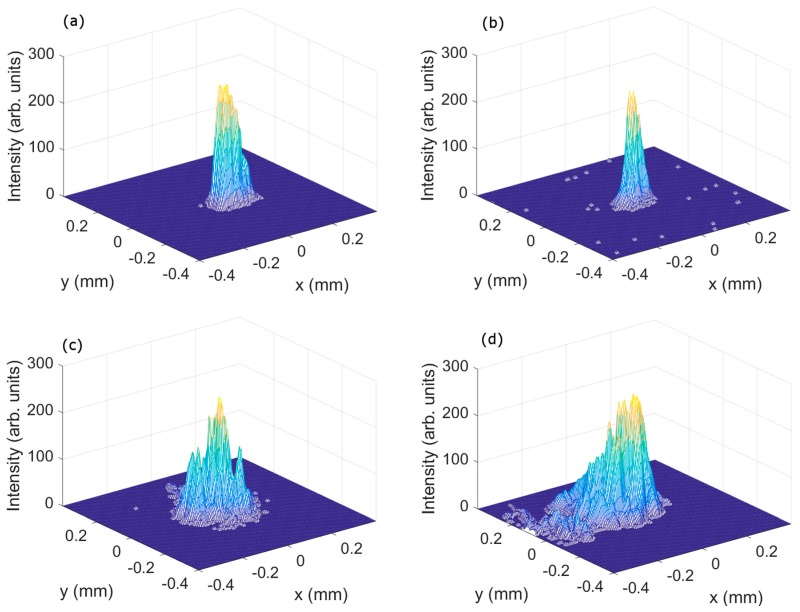
Intensity images of the HL lenses recorded (**a**) and (**b**) asymmetrically and (**c**,**d**) symmetrically, evaluating (**a**,**c**) at 473 nm and (**b**,**d**) at 633 nm.

Figure 7 shows the experimental distribution of the image plane’s intensity obtained using the CCD camera. The experimental PSF obtained from asymmetrical HLs at a wavelength of 473 nm (Figure 7a) was similar to those obtained at 633 nm (Figure 7b), as the theory predicts in Figure 6a,b. It is also observed in Figure 4a for their frequencies. In Figure 7a, the area of the experimental PSF is 0.22 × 0.16 mm^2^, and in Figure 7b is 0.20 × 0.18 mm^2^. Contrary to what would be theoretically expected, the PSF evaluated at 473 nm had a bit larger cross section than that evaluated at 633 nm. This anomaly could be due to the lack of precision in placing the CCD camera in the image plane. As would be expected, the cross section of symmetrical HLs was both larger than the asymmetrical ones. The symmetrical HLs evaluated at 473 nm wavelength (Figure 7c) were smaller than those evaluated at 633 nm (Figure 7d), as we obtained in Section 3.1 and Section 3.2.

**Figure 8:**
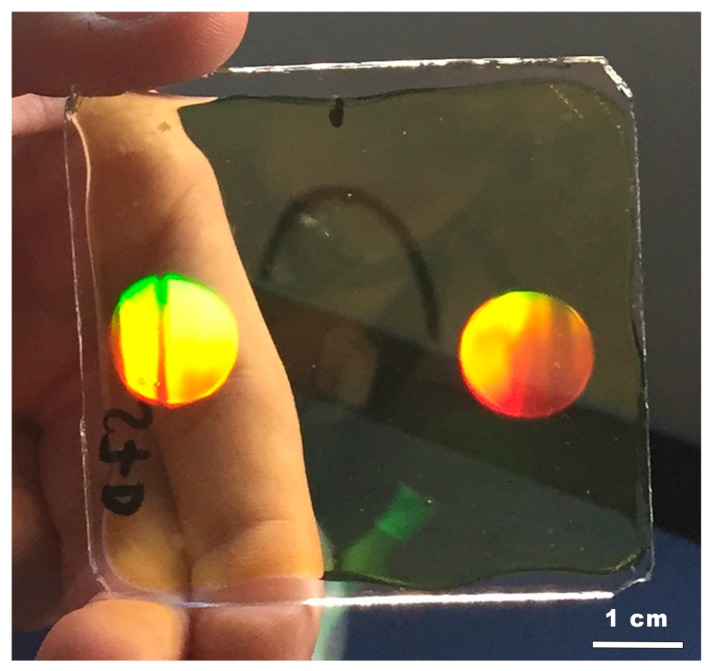
Photography of two HLs daylight-illuminated.

After evaluation, a sample with two HLs can be observed in Figure 8. The spots of the negative asymmetrical HLs diffracted the daylight in the normal direction of the sample plane. The unexposed zones were practically transparent to the sunlight.

## 4. Conclusions

Holographic lenses were stored in an environmentally friendly photopolymer. Asymmetrical and symmetrical HLs were analyzed in terms of image quality. The best results were obtained for negative asymmetrical HLs with a frequency of 14 L/mm at 473 nm wavelength for an MTF value of 0.1. The intensity distribution in the image plane was identical to the airy pattern. For this reason, the image quality was limited only by the diffraction, as can be also seen in the theoretical results. Similar results were obtained for the same negative asymmetrical HLs at 633 nm wavelength.

## Figures and Tables

**Table 1:** Recording parameters of the holographic lense (HL).

Recording Geometry	Angles	HL−	HL+
Symmetrical	θ_o, 488 nm_ = 17.1°	*d*_RL-PL_ = 240 mm	*d*_RL-PL_ =110 mm
	θ_r, 488 nm_ = −17.1°	*f*’_RL_ = 150 mm	*f*’_RL_ = 200 mm
	θ_m, 633 nm_ = −22.4°	*f*’_HL_ = −90 mm	*f*’_HL_ = 90 mm
Asymmetrical	θ_o, 488 nm_ = 0°	*d*_RL-PL_ = 240 mm	*d*_RL-PL_ = 110 mm
	θ_r, 488 nm_ = −34.2°	*f*’_RL_ = 150 mm	*f*’_RL_ = 200 mm
	θ_m, 633 nm_ = −46.8°	*f*’_HL_ = −90 mm	*f*’_HL_ = 90 mm

**Table 2:** Parameters for the evaluation of the HL.

Recording Geometry	473 nm	633 nm
Symmetrical	θ_i_ = 16.6°	θ_i_ = 22.4°
	θ_c_ = 16.6°	θ_c_ = 22.4°
	*R*_i_ = 93 mm	*R*_i_ = 70 mm
Asymmetrical	θ_i_ = 0°	θ_i_ = 0°
	θ_c_ = 33.0°	θ_c_ = 46.8°
	*R*_i_ = 93 mm	*R*_i_ = 70 mm
